# Porosity, water sorption and solubility of denture base acrylic resins polymerized conventionally or in microwave

**DOI:** 10.1590/1678-7757-2017-0383

**Published:** 2018-04-18

**Authors:** Rosana Marques Silva Figuerôa, Bruna Conterno, César Augusto Galvão Arrais, Carolina Yoshi Campos Sugio, Vanessa Migliorini Urban, Karin Hermana Neppelenbroek

**Affiliations:** 1 Universidade Estadual de Ponta Grossa, Departamento de Odontologia, Ponta Grossa, Paraná, Brasil.; 2 Universidade de São Paulo, Faculdade de Odontologia de Bauru, Departamento de Prótese e Periodontia, Bauru, São Paulo, Brasil.

**Keywords:** Polymethyl methacrylate, Polymerization, Porosity, Solubility, Sorption

## Abstract

The proper selection of polymerization cycle is important to prevent overheating of the monomer that could cause degradation, porosity and, consequently, deleterious effects on the denture base properties.

**Objective:**

This study evaluated the porosity, water sorption and solubility of acrylic resins (Vipi Cril-VC and Vipi Wave-VW) after conventional or microwave polymerization cycles.

**Material and Methods:**

Specimens (n = 10) were made and cured: 1-WB = 65°C during 90 min + boiling during 90 min (VC cycle - control group); 2-M25 = 10 min at 270 W + 5 min at 0 W + 10 min at 360 W (VW cycle); 3-M3 = 3 min at 550 W; and 4-M5 = 5 min at 650 W. Afterward, they were polished and dried in a dessicator until a constant mass was reached. Specimens were then immersed in distilled water at 37°C and weighed regularly until a constant mass was achieved. For porosity, an additional weight was made with the specimen immediately immersed in distilled water. For water sorption and solubility, the specimens were dried again until equilibrium was reached. Data were submitted to 2 way-ANOVA and Tukey HSD (α=0.05).

**Results:**

Porosity mean values below 1.52% with no significant difference among groups for both materials were observed. Resins showed water sorption and solubility values without a significant difference. However, there was a significant difference among groups for these both properties (P<0.013). The highest sorption (2.43%) and solubility (0.13%) values were obtained for WB and M3, respectively.

**Conclusions:**

The conventional acrylic resin could be polymerized in a microwave since both the materials showed similar performance in the evaluated properties. Shorter microwave cycles could be used for both the materials without any detectable increase in volume porosity.

## Introduction

Polymethyl methacrylate acrylic resin has been the material of choice for making denture bases since the beginning of the twentieth century. This material has been modified in order to improve physical and mechanical properties and facilitate the laboratory procedure using microwave polymerization, visible light curing, and vacuum pressure at low temperature curing systems^24^.

During the microwave polymerization, there is a rapid and homogeneous internal heating^6^. This alternative polymerization method has advantages such as ease and cleanliness during acrylic resin processing^12^, rapid temperature increase^17^ and, thus, a reduction in execution time^6^, minimum color change in the denture base acrylic resin and less risk of artificial teeth fracture during deflasking^16^.

The importance of the proper selection of the microwave curing cycle has been reported in order to prevent overheating of the monomer that could cause degradation^8^, porosity and consequent injury to the properties of the prosthesis^6^. According to Bafile, et al.^2^ (1991), resins designed for microwave polymerization could contain triethylene or tetraethylene glycol dimethacrylate in their composition. These dimethacrylates have low vapor pressure, allowing the polymerization to be carried out at elevated temperatures (between 100°C and 150°C) without risk of porosity, which would not occur with methyl methacrylate since it has a high vapor pressure.

The porosity is a non-desirable characteristic to the acrylic resin denture base^24^. Severe porosity can weaken the prosthesis and result in high internal stress, leading to greater vulnerability to distortion and warpage^29^. A porous surface promotes colonization of the material by oral microorganisms such as *Candida albicans* ^14^ and facilitates the retention of substances and deposition of calculus, resulting in staining and impaired aesthetic^24^ ^,^ ^30^. Water absorbed by acrylic resins during the use of prosthesis acts as a plasticizer and can result in volume changes, so the water sorption evaluation also has clinical relevance. Furthermore, residual monomer and other water soluble byproducts are released into the oral cavity and may cause tissue irritation; therefore, it is desired that these materials have low solubility^5^.

Despite the advantages of microwave polymerization, this method has gained only limited clinical acceptance, and materials specifically formulated for use in a microwave have a higher cost compared to those for conventional polymerization. Thus, aiming to overcome these disadvantages, the polymerization of a conventional resin was tested in a microwave oven, and this study evaluated the porosity and water sorption and solubility properties of the acrylic resins Vipi Cril (VC) and Vipi Wave (VW) after polymerization using experimental microwave cycles and the cycles recommended by the manufacturer. The hypotheses evaluated in this study were: 1) there would be differences regarding the porosity results between the materials in the evaluated polymerization cycles; and 2) the water sorption and solubility results would be different between the materials conventionally polymerized or cured in a microwave.

## Material and Methods

### Material

**Figure 1:**
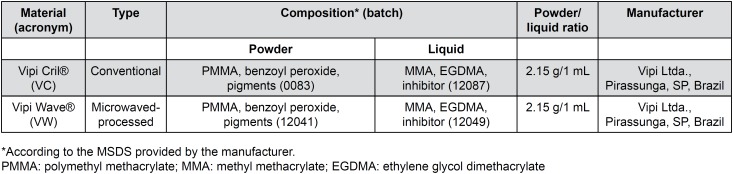
Denture base acrylic resins used in this study

The acrylic resins selected for this study are shown in Figure 1.

### Specimen preparation

For the analysis of porosity, specimens (n=10) were made in the dimensions of 50×4×2 ±0.1 mm^10^. Metal matrixes in such dimensions were molded using laboratory silicone (Zetalabor-Zhermack; Labordental, São Paulo, SP, Brazil) between two glass plates. For the analysis of water sorption and solubility, specimens (n = 10) were made in the dimensions of 50 mm in a diameter x0.5 mm thickness^10^ using metal matrixes.

**Figure 2:**
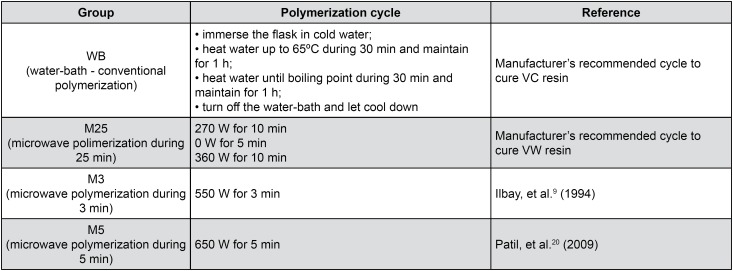
Experimental polymerization cycles evaluated in this study

The mold/matrix set or just the metal matrix was included in metallic (OGP, Bragança Paulista, SP, Brazil) or plastic (Vipi Ltda., Pirassununga, SP, Brazil) flasks using type III stone (Herodent; Vigodent Coltène SA Ind. e Com., Rio de Janeiro, RJ, Brazil). The flask was closed and remained under a load of 0.5 t in a hydraulic press (VH, Araraquara, SP, Brazil) during the setting time of the stone. After this period, the materials were handled according to the manufacturer's instructions (Figure 1) and inserted into the mold inside the flask, which was kept under a load of 1.25 t during 30 min. The specimens were then submitted to conventional (SL-155/22; Solab, Piracicaba, SP, Brazil) or microwave (MEF 41; Electrolux, Manaus, AM, Brazil) polymerization cycles as shown in Figure 2.

Then the flasks were bench cooled for 30 min and subsequently cooled in running water for 15 min before the removal of the specimens. For the porosity analysis, the specimens were polished (Aropol E; Arotec, Cotia, SP, Brazil) using silicon carbide sandpapers (#100, #240, and #600; 3M, Campinas, SP, Brazil) under constant water irrigation. For the water sorption and solubility specimens, a minicutt carbide bur (Edenta; Bergün, Graubünden, Switzerland) was used to remove irregularities of their edges. The specimens were then visually examined, and only those with minimal porosity were used^10^.

### Porosity

Porosity was related to the amount of absorbed water by each specimen after storing in distilled water at 37°C. The samples were weighed on a digital analytical balance (four decimal places AW220; Shimadzu do Brazil, São Paulo, SP, Brazil) in two stages. In the first one, the specimen was daily weighed after a storing period in a desiccator (Vidrolabor, São Paulo, SP, Brazil). In the second one, the specimen was also daily weighed after a storing period in distilled water in an oven (New Instruments, Piracicaba, SP, Brazil) at 37°C. The final record of weighing for dry and wet specimens was carried out at the time that they reached a stable mass, evidenced after stabilization in a milligram scale.

The porosity was analyzed using the gravimetric method based on Archimedes’ principle^2^ ^,^ ^4^ ^,^ ^18^ ^,^ ^21^ and, therefore, an additional weighing was performed after each stage with the specimen immersed in distilled water.

Thereafter, the porosity was calculated according to the following equations:

[1] Vs dry=(md−md')/ρwater

[2] Vs wet=(mw−mw')/ρwater

[3] Porosity%=100×(Vsdry−Vswet)/Vsdry

where: Vs *dry* (mL) is the volume of the dry specimen; m_d_ (g) is the mass of the dry specimen recorded in air; m_d_’ (g) is the mass of the dry specimen recorded with the specimen immediately immersed in water; ρ_water_ (g/mL) is the density of water; Vs *wet* (mL) is the volume of the wet specimen; m_w_ (g) is the mass of the wet specimen recorded in air; and m_w_’ (g) is the mass of the wet specimen recorded with the specimen immediately immersed in water.

### Water sorption and solubility

Specimens were subjected to a drying process in order to achieve a constant weight. So, they were kept in a vacuum desiccator and daily weighed on a digital analytical balance until the difference between sequential weight measurements was less than 0.5 mg. After obtaining the constant mass, specimens were stored in distilled water at 37°C and were also daily weighed until stabilization, but always after careful drying with absorbent paper.

To calculate the water sorption and solubility, specimens passed again by the above drying process. The water sorption and solubility percentages were calculated using the following equations^15^:

[4] $$\%\text{Sorption}=100\times (m_{2}-m_{3})/m_{1}$$

[5] $$\%\text{Solubility}=100\times (m_{1}-m_{3})/m_{1}$$

where: m_2_ is the mass (mg) of the specimen after immersion in water; m_3_ is the mass (mg) of the specimen after the second drying; and m_1_ is the mass (mg) of the specimen after the first drying.

### Statistical analysis

The results of porosity, water sorption and solubility (%) were analyzed using two-way ANOVA (“material” and “polymerization cycle” factors) and followed by Tukey HSD test (α=0.05) using a personal software (IBM SPSS 19; SPSS Inc., IBM Company, Armonk, NY, USA). *Post-hoc* power analysis was performed for statistical analyses of all data using the personal statistical software (IBM SPSS 19).

## Results

For the number of specimens used to evaluate the porosity, water sorption and solubility properties (n = 10) of denture base acrylic resins, this study showed adequate power for both factors “material” and “polymerization cycle” for the three properties (100%, 85.2%, and 80.4%, respectively; α=0.05).

**Figure 3:**
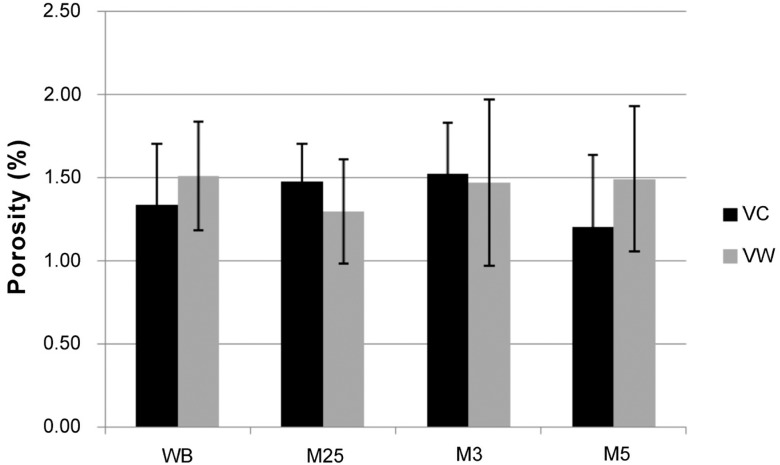
Mean and standard deviation porosity values (%) for VC and VW resins polymerized in water-bath and microwave cycles

The porosity results are shown in Figure 3. There were no significant differences (*P*>0.05) between the evaluated polymerization cycles for both the materials, and the observed mean values were less than 1.52%.

**Figure 4:**
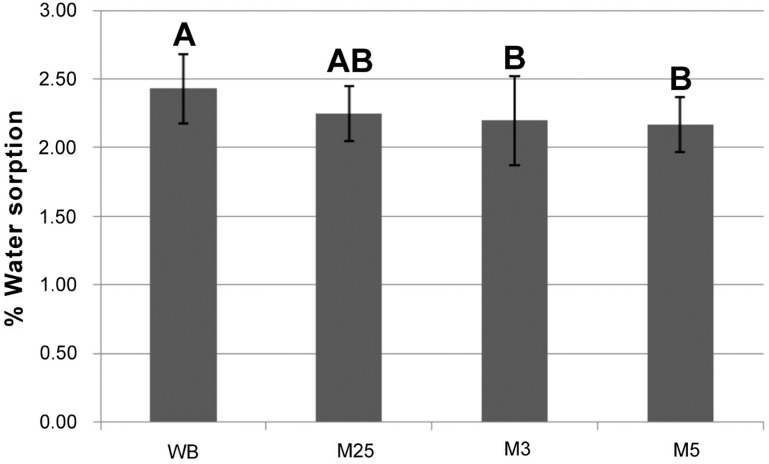
Mean and standard deviation water sorption values (%) obtained polymerizing in water-bath and microwave cycles regardless of the material. Identical capital letters indicate values with no statistically significant difference (P>0.05)

**Figure 5:**
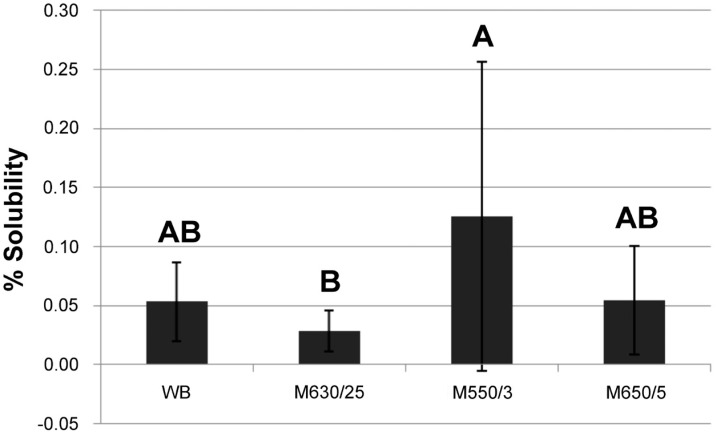
Mean and standard deviation solubility values (%) obtained polymerizing in water-bath and microwave cycles regardless of the material. Identical capital letters indicate values with no statistically significant difference (P>0.05)

For the water sorption and solubility analyses, there was no significant difference between the acrylic resins (*P*>0.05). However, significant differences were noted (*P*<0.013) among the experimental cycles for both the properties (Figures 4 and 5). According to Figure 4, the highest water sorption mean values were obtained in the WB cycle (2.43%); intermediate values in the M25 cycle (2.25%); and the lowest values in the experimental microwave cycles M3 (2.20%) and M5 (2.17%). Figure 5 shows that the M3 cycle resulted in the highest mean solubility values (0.13%); intermediate values were observed in the M5 and WB cycles (0.05%); and the lowest values in the M25 cycle (0.03%).

## Discussion

The first hypothesis evaluated in this study, in which “there would be differences regarding the porosity results between the materials in the evaluated polymerization cycles”, was not accepted, since no significant differences were observed between the acrylic resins conventionally polymerized in the water- bath or processed in a microwave.

A variety of methods have been used to measure the porosity of acrylic resins using microscopy, mercury porosimetry and the classic method by measuring the weight of the specimen before and after immersion in water, the volume of the specimen and density of acrylic resin and the water and air confined in the pores^2^ ^,^ ^4^ ^,^ ^30^. According to Yannikakis, et al.^30^ (2002) the classic method is more objective but does not provide detailed information on the size and location of the pores. However, this study aimed not to describe the pores but objectively compare the polymerized groups according to the manufacturer's instructions or experimentally.

The porosity is a complex phenomenon attributed to a variety of factors depending on the laboratory technique and partially on the conjunction of the polymerization method and material^13^ ^,^ ^30^. All factors related to the laboratory technique that would influence the porosity results; the proportioning and incorporation of the powder to the liquid; and the pressing, cooling of the flask and polishing time were controlled^19^. The results of other studies^2^ ^,^ ^9^ ^,^ ^19^ also found no significant differences in porosity values when the thicknesses of the specimens polymerized in several cycles were around 3 mm. Also, Yannikakis, et al.^30^ (2002) reported that it is possible to polymerize a conventional resin in a microwave when its thickness is equal or less than 3 mm, since this thickness is representative of dimensions commonly used for making denture bases^30^. With regard to materials, a similarity in their composition could be noted in Figure 1 that shows the information provided by the manufacturer. Thus, it is possible to suppose that there was no difference between the results of porosity due to standardization in the laboratory technique, the thickness of the specimens and the similar composition of the materials.

According to the American Dental Association specifications, there should be no bubbles or voids in the denture base polymer when viewed without magnification. Furthermore, porosity values above 11% have been associated with reduced mechanical properties, impaired appearance and retention of fluids and microorganisms^11^, and levels lower than this one may be considered clinically acceptable^24^. In this study, visible pores and bubbles were not observed and all porosity values were less than 1.52%. Since handling errors were eliminated, the evaporation of the monomer due to the high temperature (external heat + exothermic reaction) is then the most likely cause of the detected internal porosity^30^.

The hypothesis that “the water sorption and solubility results would be different between the materials conventionally polymerized or cured in microwave” was partially proven as there was no difference between the tested materials, but the polymerization cycles resulted in different values for water sorption and solubility.

There are two techniques to quantify the water sorption. The technique recommended by ADA N° 12^1^ takes into consideration the specimen weight after saturation (m_2_) minus the initial dry weight (m_1_). This technique assesses the relative sorption of specimens. The technique described by Kazanji and Watkinson^15^ (1988) measures the weight after saturation (m_2_) minus the final dry weight (m_3_) and not the initial dry weight. This technique is considered the sorption of the polymer network as it takes into account the absorbed liquid minus the liquid released by the material. In the evaluation of water sorption and solubility, the Kazanji and Watkinson technique^15^ (1998) is more representative (data in percent) and has less variable (surface area, results in mg/cm^2^) in the calculations^7^. In this study, we used the technique recommended by Kazanji and Watkinson^15^ (1998).

The fact that there was no difference between the materials for water sorption and solubility, as well as for porosity, is due to their similar composition as mentioned earlier. The water sorption mean values were between 2.17% and 2.43%. These values have been considered appropriate since the acrylic resin initially desiccated becomes saturated after sorption of approximately 2% of water^26^. The solubility values found in this study (between 0.0065% and 0.4715%) were within the range of values observed in another study (between −0.417% and 0.815%) that evaluated the effect of chemical or mechanical polishing on the solubility of Vipi Cril resin. This large variation, mainly observed in the solubility data, can be attributed to small measured variations in weight^7^. Lower values of solubility than water sorption are due to slow elution of the residual monomer in comparison to water diffusion^26^. This is because the dissolution of the monomer is 0.5 to 1.7x10^−2^ times smaller than the water sorption of the resin based on polymethyl methacrylate^25^.

The acrylic resins contain polar carbonyl groups that therefore attract water molecules^26^. Water molecules, in turn, diffuse between the polymer intermolecular gaps separating them slightly^22^ and gradually infiltrate deeper into the resin^26^. The most important consequence of water sorption is the dimensional change^22^, resulting in change in the vertical dimension of occlusion previously determined. In contrast, it has been reported that the water sorption partially compensates the polymerization shrinkage of heat-polymerized acrylic resin, and after reaching the saturation, the prosthesis should fit better than immediately after processing since there has been no change in paraprosthetic tissues^28^.

The highest water sorption values observed in the water-bath conventional polymerization group may be related to a low residual monomer content^5^ and a higher degree of conversion. The use of a final temperature for at least 30 min in the water-bath polymerization cycles has led to reduced residual monomer content^27^. This study evaluated the water-bath polymerization recommended by the manufacturer that was included at the end of the cycle a period of 1 h in boiling for both resins. The reduction of the residual monomer is related to the temperature increase at the end of the polymerization cycle, which results in increased mobility of the molecular chains, thus facilitating the conversion of monomer to polymer^23^. Similarly, the lowest water sorption results observed in M3 and M5 groups can be associated with a low degree of conversion values. Moreover, the highest solubility values found in the M3 group can be due to a higher residual monomer content^20^ because it has been related to higher levels of solubility of acrylic resins^3^ ^,^ ^5^.

This study evaluated only two brands of heat- polymerized denture base acrylic resins. Additional studies evaluating physical, mechanical and biological properties and the material behavior after aging conditions are needed to decide if faster microwave polymerization cycles can be used to cure conventional acrylic resins.

## Conclusions

Within the limitations of this *in vitro* study, it can be concluded that the conventional acrylic resin Vipi Cril could be polymerized in a microwave, since both the materials behaved similarly in the evaluated properties. Shorter microwave polymerization cycles could be used for both the materials without any detectable increase in volume porosity. The lowest water sorption and the highest solubility values were detected by using the microwave polymerization at 550 W for 3 min.
